# Improved Scoring of the Center for Epidemiologic Studies Depression Scale – Revised: An Item Response Theory Analysis

**DOI:** 10.1007/s10862-024-10155-y

**Published:** 2024-07-16

**Authors:** Christian A. L. Bean, Sophia B. Mueller, George Abitante, Jeffrey A. Ciesla, Sun-Joo Cho, David A. Cole

**Affiliations:** 1 Department of Psychology and Human Development, Vanderbilt University, Nashville, TN USA; 2 Department of Psychological Sciences, Kent State University, Kent, OH USA

**Keywords:** Center for epidemiologic studies depression scale – revised, CESD-R, Item response theory, Psychometrics, Cutoff score, Depression

## Abstract

The Center for Epidemiologic Studies Depression Scale – Revised (CESD-R) is a popular self-report screening measure for depression. A 20-item questionnaire with scores ranging from 0 to 4 for each item, the CESD-R can produce total scores ranging from 0 to 80. However, the typical scoring protocol for the CESD-R restricts the range of possible scores to between 0 and 60 to retain the same range and clinical cutoff scores as the original CES-D. Despite the widespread adoption of this scoring approach, the psychometric impact has never been systematically examined. In an undergraduate and community adult sample (*n* = 869), item response theory analyses indicated that scoring the CESD-R with all 5 response options (CESD-R_5opt_) provided nearly twice as much information about a person’s latent depression for individuals with high levels of depression than did scoring the CESD-R with 4 response options per item (CESD-R_4opt_). The CESD-R_5opt_ retained the strong reliability and factor structure of the CESD-R_4opt_ and was more sensitive to individual differences for participants at high levels of depression compared to the CESD-R_4opt_. Results provide preliminary evidence that researchers and clinicians should score the CESD-R using the full 0-to-80 scale and a clinical cutoff score of 29.

**Supplementary Information:**

The online version contains supplementary material available at 10.1007/s10862-024-10155-y.

The Center for Epidemiologic Studies Depression Scale (CES-D; Radloff, 1977) is one of the most popular depression self-report screening measures, having received 65,070 citations according to Google Scholar as of March 7, 2024. In 2004, a revised version of the measure, the Center for Epidemiologic Studies Depression Scale – Revised (CESD-R; Eaton et al., 2004), was published and featured an additional response option for each item. Despite this extra response option, the CESD-R was accompanied by a change in its scoring protocol to keep the range of possible scores equivalent to that of the earlier CES-D. This scoring change was implemented to prevent users from having to adopt new clinical cutoff scores when they shifted to the new measure. Unfortunately, this change also paved the way for a potential psychometric liability in the CESD-R by reducing its sensitivity to individual differences among people with high levels of depression – the very people of greatest interest to many depression researchers and clinicians who use the CESD-R. Consequently, the overarching goals of the current study were to quantify this loss of information, recommend a solution via an improved scoring protocol, and test whether new clinical cutoffs may be needed.

The original CES-D was a 20-item questionnaire that asked respondents to indicate how often they have experienced each symptom (i.e., item) over the past week on a 4-point scale from 0 (“rarely or none of the time (less than 1 day)”) to 3 (“most or all of the time (5–7 days)”). Item scores were summed to create a total score that ranged from 0 to 60, with greater values indicating more severe depressive symptoms. The originally proposed clinical cutoff score for the probable presence of clinically significant depression was 16 (Radloff, 1977). Over the decades since its publication, various alternative cutoff scores have been proposed such as 21 (Henry et al., 2018) and 25 (Haringsma et al., 2004), although the most conservative and commonly used clinical cutoff score of 27 has been recommended by multiple independent research groups (Gotlib et al., 1995; Kagee et al., 2020; Schulberg et al., 1985; Zich et al., 1990).

Despite its widespread use, the CES-D was developed before the introduction of the Diagnostic and Statistical Manual of Mental Disorders (DSM)-III (APA, 1980) and therefore did not reflect modern diagnostic criteria for depression. Specifically, the CES-D did not include items assessing anhedonia, psychomotor agitation/retardation, or suicidal ideation but did include eight items that are no longer diagnostically relevant to major depressive disorder (such as the items “I felt fearful” and “People were unfriendly”; Eaton et al., 2004). Furthermore, the CES-D also only asked people to respond about the past week, also at odds with the DSM-IV (APA, 1994) in which major depressive disorder symptoms are assessed over a two-week period. To overcome these limitations, the primary goal in revising the CES-D was to update its structure and content to map more accurately onto diagnostic criteria for major depressive disorder as per the DSM-IV (APA, 1994). To do this, item content was updated and a new response option was added. Whereas the most severe response option on the CES-D for any item was “5–7 days” over the past week, the CESD-R added a fifth option, “nearly every day for 2 weeks,” which was given a score of 4 and corresponded better with DSM-IV diagnostic criteria.

The CESD-R is the most recent version of the CES-D and retains the 20-item structure of its predecessor. With the inclusion of the additional response option, scores on the CESD-R can range from 0 to 80. However, the developers of the CESD-R recommended that a score of 4 on any item be converted to a score of 3 when the total raw score is calculated, so that actual scores on the CESD-R range from 0 to 60, just as they did for the CES-D (Eaton et al., 2004). The assumption was that this would allow clinical cutoff scores for the CES-D to be directly applied to the CESD-R and allow the large evidence base of the CES-D to support the validity of the CESD-R. As a result, response options of 3 (symptoms occurring 5–7 days over the past two weeks) and 4 (symptoms occurring nearly every day for the past two weeks) are treated identically for the purposes of scoring the CESD-R (with both response options being assigned scores of 3).

The CESD-R has been validated in various populations (Kimong et al., 2023; Van Dam & Earleywine, 2011) and demonstrated configural, metric, and scalar measurement invariance between biological males and females in a large, racially diverse undergraduate student sample (Sriken et al., 2023). Surprisingly, however, the psychometric impact of collapsing the two most severe response options on the CESD-R and restricting the range of possible total scores has never been systematically examined.

In the current study, we sought to examine the effects of combining response options on the CESD-R using item response theory (IRT) in a large, combined undergraduate and community adult sample. The CESD-R was completed once per participant but scored two ways: first using the conventional 0–60 scoring with 4 response options per item (hereafter known as the CESD-R_4opt_ for simplicity) and again using the full-information 0–80 scoring with 5 response options per item (i.e., the CESD-R_5opt_). We conducted IRT analyses to evaluate psychometric difference between the CESD-R_4opt_ and CESD-R_5opt_. Specifically, we examined the relative efficiency of the test and item information of underlying level of depressive symptoms to determine whether the CESD-R_5opt_ provides more information about a person’s depressive symptomatology than the CESD-R_4opt_ at the test and item levels. We also examined differences between item thresholds in an item response model, as separated thresholds would suggest that a response of 4 (nearly every day over the past two weeks) requires substantively greater levels of latent depression than a response of 3 (5–7 days over the past two weeks), which would indicate that these response options represent meaningful differences in one’s underlying level of depressive symptoms.

We hypothesized that the CESD-R_5opt_ would show greater relative efficiency and that separated item thresholds in an item response model would be observed between response options 3 and 4. To examine the effects of scoring choice on general psychometric properties of the CESD-R, exploratory analyses tested potential differences in factor structure and internal consistency reliability between the CESD-R_4opt_ and CESD-R_5opt_. Finally, we conducted preliminary comparisons of percentile scores for the CESD-R_4opt_ and CESD-R_5opt_ to estimate the scaling differences and ascertain whether different clinical cutoffs would be needed for the CESD-R_5opt_.

## Method

### Participants

The data analytic sample was a combination of data from two sources. We used an online research panel through Qualtrics Survey Panels to collect Sample 1 (*n* = 516), a general community adult sample, between October and November 2023. Eligibility criteria were proficiency in English, residing in the United States, and being at least 18 years old. All participants in Sample 1 were monetarily compensated and also completed other measures extraneous to the current investigation. Mean completion time for all measures was 23.68 min (*SD* = 31.77 min, median = 16.43 min). To ensure data quality, participants who completed all surveys in 8 min or less (approximately half the median completion time) were excluded from the final sample and all analyses. Additionally, three attention check items were included (e.g., “Please select five to seven days”) and participants who provided incorrect answers to any of these items were also excluded. Sample 2 (*n* = 363) was made up of undergraduate students at a large, midwestern public university who completed the CESD-R between March 2018 and July 2022 (see Bean & Ciesla, 2021, 2023; and Bean et al., 2023 for additional details on data collection procedures). Ten participants from Sample 2 did not complete all items on the CESD-R and were removed from all analyses. Informed consent was provided by all participants in both samples. This study received ethics approval from the institutional review board of the affiliated universities.

To generate a large sample of people spanning the full range of depression, we combined the two samples. Combining samples was justified by differential item functioning (DIF) analysis with the ‘lordif’ R package (Choi et al., 2011) and McFadden’s pseudo effect sizes. We used non-uniform, uniform, and total DIF to compare models. Effect sizes across all DIF comparisons ranged from 0.000 to 0.029 (refer to the online supplement for results of likelihood ratio tests in Table S1, McFadden’s pseudo in Table S2, and graphical DIF in Figure S1). Based on guidelines recommended by Cohen (1988), all effects were of negligible size. This result means that all items functioned similarly across both samples. As a result, the two data sets can be combined for further analyses. Demographic characteristics of the combined data set (*n* = 869) are shown in Table 1. Most participants were White, non-Hispanic or Latino females. The average age of participants was 36.49 years (*SD* = 19.42).

**Table 1:** Demographic information

Demographic	*n* (%)
Gender	
Male	319 (36.71)
Female	532 (61.22)
Other	18 (2.07)
Race	
Asian	34 (3.91)
Black or African American	110 (12.66)
Native American or Alaskan Native	17 (1.96)
Native Hawaiian or Other Pacific Islander	2 (0.23)
White or European American	667 (76.75)
Other	43 (4.95)
Not Reported	8 (0.92)
Ethnicity	
Hispanic or Latino	100 (11.51)
Not Reported	2 (0.23)

### Data Analysis Plan

Parallel analysis and exploratory factor analysis (EFA) were conducted to examine the number of factors and their structures for the subsequent IRT analyses. Additionally, we evaluated several test-level reliability metrics (IRT marginal reliability, McDonald’s omega, and coefficient alpha). We used IRT to evaluate the difference between the CESD-R_4opt_ and CESD-R_5opt_. First, we compared the two polytomous item response models with and without item discriminations to check whether item discriminations are needed to characterize items adequately: a partial credit model (PCM; Masters, 1982) and a graded response model (GRM; Samejima, 1969). In addition, the item fit of a selected item response model was evaluated. We examined relative efficiency of the test information obtained from the selected item response model to determine whether using the CESD-R_5opt_ provided more information on depressive symptoms across the entire test items. Similarly, we evaluated relative efficiency of the item information to determine whether the CESD-R_5opt_ items provided more information compared to the CESD-R_4opt_ items. We examined the order and separation of item thresholds under a selected item response model to determine whether thresholds corresponding to the CESD-R_4opt_ scoring versus CESD-R_5opt_ were different. Separated thresholds would suggest that substantively different levels of latent depression were required for a response of 4 versus 3. We used item characteristic curves to confirm visually if thresholds were separated. Separation between the curves of the last two thresholds indicates a difference in the two response options that have traditionally been collapsed. We used *Mplus version 8.10* (Muthén & Muthén, 1998–2024) to fit bifactor EFAs (Jenrich & Bentler, 2011; 2012). Regarding other analyses, we used ’EFAutilities’ for parallel analysis (Zhang et al., 2016) for EFA and ‘mirt’ (Chalmers, 2012) for IRT analyses in *R version 4.2.2* (R Core Team, 2022).

## Results

### Preliminary Analyses

#### Exploratory Factor Analysis

Parallel analysis indicated the presence of a dominant general factor and several minor factors for the CESD-R_4opt_ and CESD-R_5opt_ (see Figures S2a and S2b in the online supplement). The ratios of the first to second eigenvalue were 10.32 and 10.54 for the CESD-R_4opt_ and CESD-R_5opt_, respectively, which suggests the presence of a dominant general factor (Reise et al., 2010). To assess whether the minor factors can be ignored, we considered a bifactor EFA to extract a dominant general factor and minor factors. We then compared the factor loadings that emerged for the 1-factor solution with those obtained from the dominant general factor in the bifactor EFA. Model fit was slightly better for the bifactor EFAs; however, factor loadings were similar between the 1-factor model and bifactor EFAs. The correlations between the factor loadings obtained from the 1-factor model and those of a dominant general factor obtained from the bifactor EFA controlling for *one* minor factor were 0.93 for the CESD-R_4opt_ and 0.94 for the CESD-R_5opt_. When controlling for *two* minor factors, the correlations were 0.85 and 0.86 for the CESD-R_4opt_ and CESD-R_5opt_, respectively. These results indicated that we could ignore the minor factors initially identified in parallel analysis. Factor loadings of the 1-factor EFA (with oblimin rotation) for both scoring options are shown in Table 2. The factor loadings were extremely similar for both scoring methods, although slightly higher for most items when full scoring was used. Furthermore, item parameter estimates and latent variable scores from a unidimensional item response model are typically only minimally affected by other factors when a dominant general factor is present (e.g., Ansley & Forsyth, 1985; Reckase, 1979; Way et al., 1988). Based on the sensitivity analysis of minor factors using the bifactor EFA and their negligible effects on IRT estimation, unidimensionality was assumed for subsequent IRT analyses.

**Table 2:** Exploratory factor analysis loadings for a 1-factor solution between scoring methods

Item	CESD-*R*_4opt_	CESD-*R*_5opt_
1. My appetite was poor.	0.589	0.592
2. I could not shake off the blues.	0.839	0.847
3. I had trouble keeping my mind on what I was doing.	0.765	0.761
4. I felt depressed.	0.840	0.845
5. My sleep was restless.	0.602	0.616
6. I felt sad.	0.824	0.829
7. I could not get going.	0.804	0.808
8. Nothing made me happy.	0.792	0.789
9. I felt like a bad person.	0.723	0.735
10. I lost interest in my usual activities.	0.804	0.810
11. I slept much more than usual.	0.477	0.498
12. I felt like I was moving too slowly.	0.693	0.699
13. I felt fidgety.	0.643	0.648
14. I wished I were dead.	0.608	0.626
15. I wanted to hurt myself.	0.522	0.546
16. I was tired all the time.	0.714	0.720
17. I did not like myself.	0.782	0.785
18. I lost a lot of weight without trying to.	0.334	0.356
19. I had a lot of trouble getting to sleep.	0.618	0.626
20. I could not focus on the important things.	0.788	0.789

#### Test-Level Reliability

We estimated the reliabilities of both scoring methods. Cronbach’s alpha was 0.95 for both versions of the measure. Similarly, McDonald’s omega was 0.95 for the CESD-R_4opt_ and CESD-R_5opt_. IRT marginal reliability was 0.92 for the CESD-R_4opt_ and 0.93 for the CESD-R_5opt_. Reliabilities of the CESD-R_4opt_ and CESD-R_5opt_ were approximately the same but slightly higher for the CESD-R_5opt_ using IRT marginal reliability.

### Model Selection and Item Fit

For the CESD-R_5opt_, both the Akaike Information Criterion (AIC; Akaike, 1973) and Bayesian Information Criterion (BIC; Schwarz, 1978) were lower for the GRM (AIC = 35198.28, BIC = 36237.57) compared to the PCM (AIC = 36036.90, BIC = 36985.60), indicating that the GRM was a better fit for the CESD-R_5opt_. Likewise, for the CESD-R_4opt_, the AIC and BIC were lower for the GRM (AIC = 31778.70, BIC = 32722.63) compared to the PCM (AIC = 32553.34, BIC = 33406.69). The item-fit index root mean square error of approximation (RMSEA) for both versions of the CESD-R was less than 0.05 for all items (see Table S3 in the online supplement). This result suggests that items fit well to the data under the selected GRM model.

### Item Response Theory (IRT) Analyses

#### Test Information

**Fig. 1:**
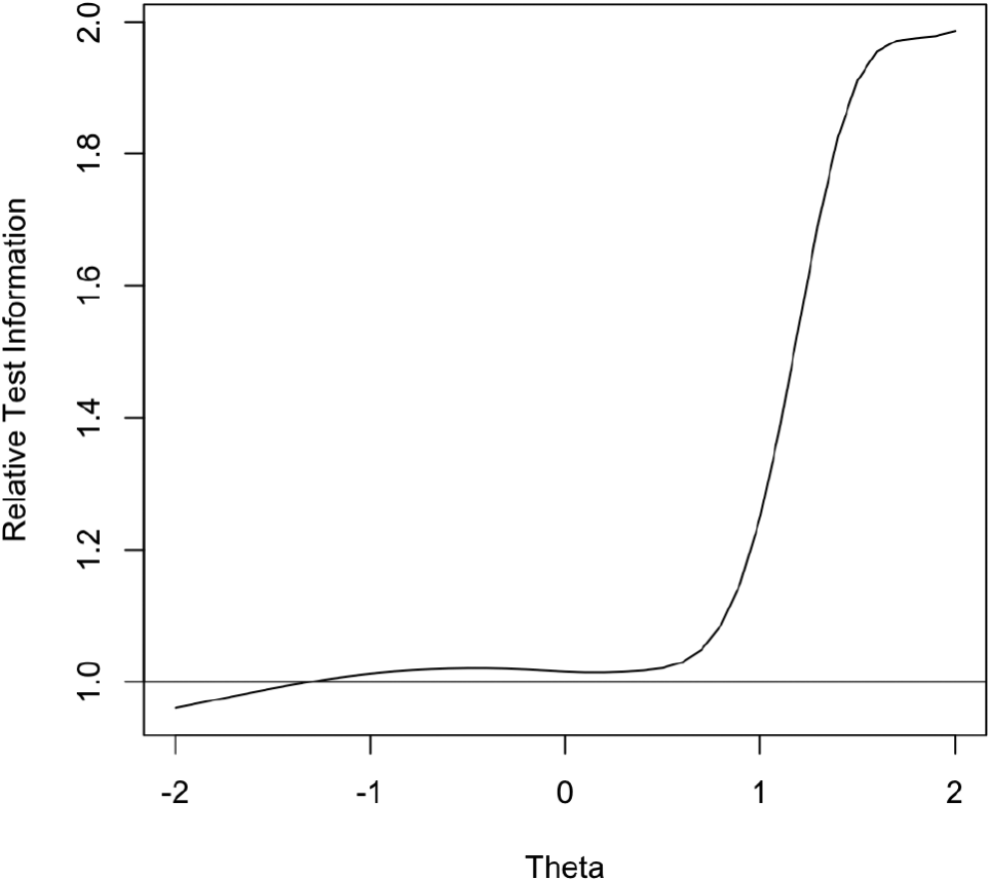
Relative efficiency curve describing the ratio of test information for CESD-R_5opt_ to test information for CESD-R_4opt_

Test information based on GRM results indicates the amount of information that the test provides at different levels of latent depression denoted by “theta”. To evaluate whether the CESD-R_4opt_ and CESD-R_5opt_ options provided equivalent information, we used relative efficiency to compare test information from the CESD-R_4opt_ and CESD-R_5opt_. The relative efficiency is defined as the ratio of test information for CESD-R_5opt_ to that of CESD-R_4opt_. The relative efficiency of 1 means that both CESD-R_4opt_ and CESD-R_5opt_ provide an equivalent amount of test information, indicating that neither test is more efficient at measuring depression over the range of levels assessed. The relative efficiency curve in Fig. 1 demonstrated that *for low to moderate theta values*, the test information from both scoring options was approximately the same. However, *at higher theta values* (> 1.0), test information was substantially greater for the full-scoring option. The information gained when using the CESD-R_5opt_ was approximately 1.5 to 2 times greater compared to the CESD-R_4opt_. This increase in test information indicated that using the full scoring option provided substantially more information for individuals with higher levels of depression.

#### Item Information

**Fig. 2:**
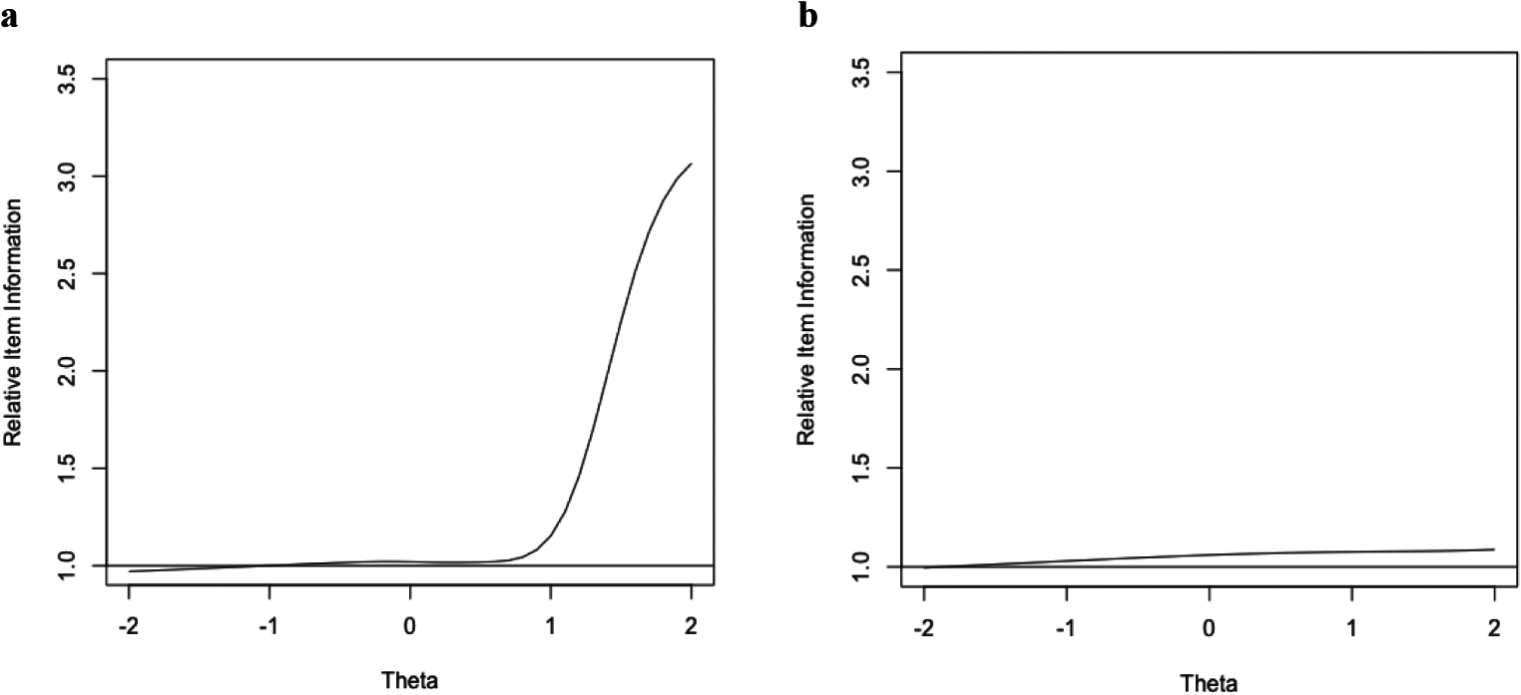
**a** Relative Efficiency Curve Describing the Ratio of Item Information for CESD-R_5opt_ to that of CESD-R_4opt_ for Item 10. **b**: Relative Efficiency Curve Describing the Ratio of Item Information for CESD-R_5opt_ to that of CESD-R_4opt_ for Item 18

Item information indicates the amount of information that each item provides at different levels of theta. Relative efficiency estimates for each item are available in Figure S3 in the online supplement. Most items had a pattern confirming the conclusions from the relative efficiency based on the test information results. For example, in the relative efficiency of item 10 (see Fig. 2a), values near 1.0 on the left side of the graph signify that the two scoring methods generated the same amount of information at low levels of depression; however, the larger values on the right indicate that the full-scoring method generated much more information at higher levels of depression. Only one item showed a negligible difference in information across the two scoring options (item 18: “I lost a lot of weight without trying to”; see Fig. 2b).

#### Item Thresholds and Item Characteristic Curves

**Fig. 3:**
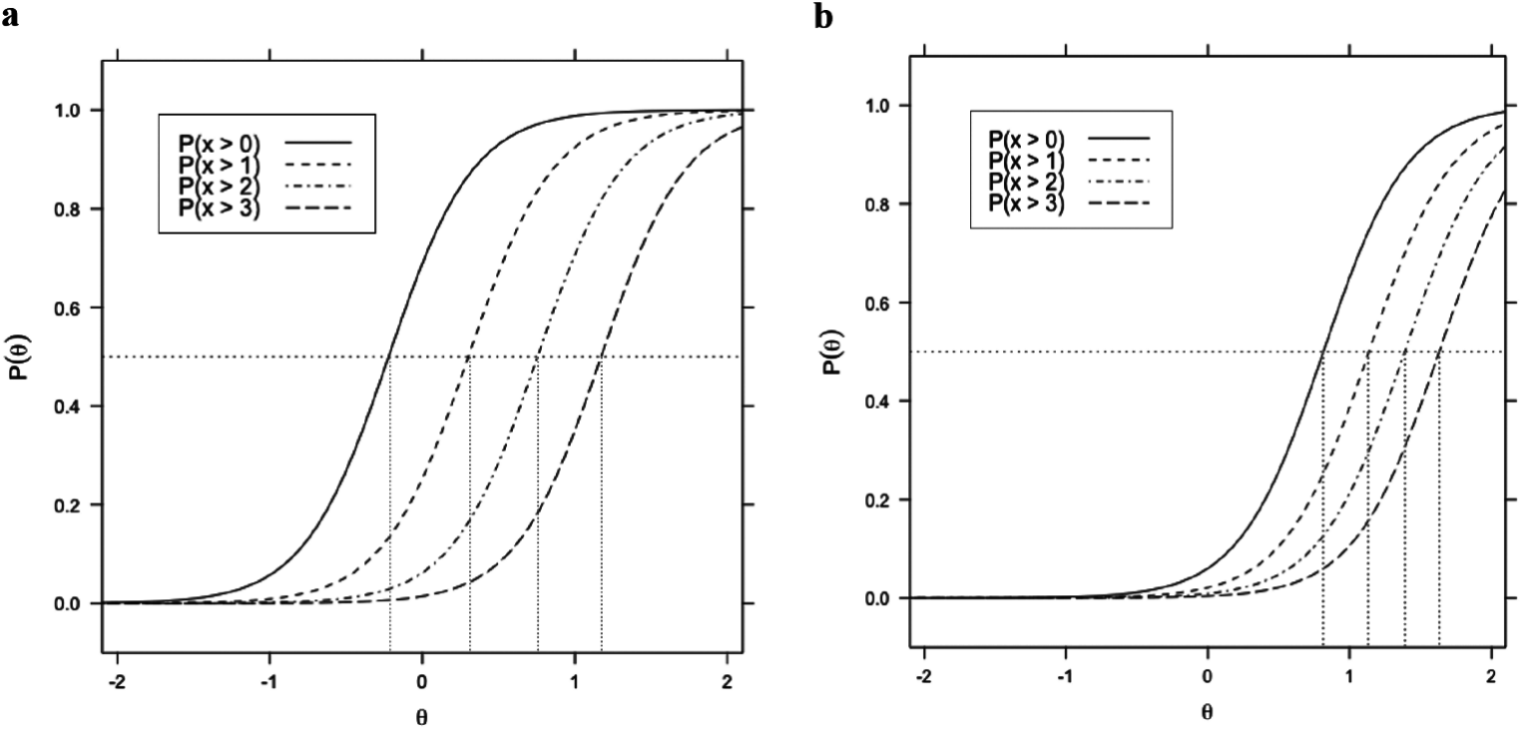
**a** Item characteristic curve for Item 3 for CESD-R_5opt_. *Note*: P(x > 0) indicates the probability of having a score above 0; P(x > 1) indicates the probability of having a score above 1; P(x > 2) indicates the probability of having a score above 2; P(x > 3) indicates the probability of having a score above 3. Threshold estimates for Item 3 (-0.221, 0.302, 0.756, 1.173) correspond to the “theta” points at which each probability equals 0.5. **b** Item characteristic curve for Item 14 for CESD-R_5opt_. *Note* P(x > 0) indicates the probability of having a score above 0; P(x > 1) indicates the probability of having a score above 1; P(x > 2) indicates the probability of having a score above 2; P(x > 3) indicates the probability of having a score above 3. Threshold estimates for Item 14 (0.816, 1.126, 1.389, 1.632) correspond to the “theta” points at which each probability equals 0.5

To evaluate the separation between response options 3 and 4 in the full-scoring version of the CESD-R, we examined GRM threshold estimates and their standard errors. Using GRM with the full scoring option, each CESD-R item has four cumulative thresholds. Threshold 1 compares response option 0 to responses 1–4. Threshold 2 compares response options 0 and 1 to responses 2–4. Threshold 3 compares response options 0–2 to responses 3 and 4. Threshold 4 compares response options 0–3 to response option 4. Thresholds indicate the theta value at which the probability of responding to that response category or higher is 0.50. For instance, an estimate for threshold 3 would indicate the theta value where the probability of responding to a score above 2 is 0.50. In comparison, the threshold 4 estimate indicates the theta value when the probability of responding to a score above 3 is 0.50. A separation between thresholds 3 and 4 on any item suggests that moving from a score of 3 to a score of 4 corresponds to a meaningful increase in latent depression. Across all items, thresholds were ordered and distinctly separated (see Table 3). The average separation between thresholds 3 and 4 across items was 0.35 (*SD* = 0.08). Separation of threshold estimates was visually evident in the item characteristic curves (ICCs). The degree of separation between thresholds 3 and 4 varied across items, but all thresholds were substantially separated. No curves overlapped. For example, the ICC for items 3 and 14 in Fig. 3a and b, respectively, are representative, showing a clear separation between thresholds. ICCs for all items are available in Figure S4 in the online supplement.

**Table 3:** Item parameter estimates and standard errors (SEs) of graded response model

Item	Item Discrimination	Threshold 1		Threshold 2		Threshold 3		Threshold 4	
		Estimate	SE	Estimate	SE	Estimate	SE	Estimate	SE
1. My appetite was poor.	2.110	0.015	0.039	0.641	0.045	1.183	0.066	1.524	0.086
2. I could not shake off the blues.	5.195	0.012	0.021	0.527	0.023	0.885	0.028	1.208	0.037
3. I had trouble keeping my mind on what I was doing.	3.593	-0.221	0.029	0.302	0.026	0.756	0.032	1.173	0.044
4. I felt depressed.	5.052	-0.005	0.022	0.463	0.022	0.718	0.025	0.970	0.031
5. My sleep was restless.	2.058	-0.403	0.048	0.402	0.040	0.935	0.055	1.306	0.073
6. I felt sad.	4.517	-0.275	0.026	0.345	0.023	0.734	0.027	1.144	0.037
7. I could not get going.	3.991	-0.097	0.026	0.483	0.026	0.896	0.032	1.229	0.043
8. Nothing made me happy.	4.396	0.234	0.023	0.671	0.027	0.991	0.033	1.263	0.043
9. I felt like a bad person.	3.521	0.274	0.026	0.679	0.031	1.047	0.042	1.356	0.054
10. I lost interest in my usual activities.	4.223	0.103	0.024	0.577	0.026	0.958	0.033	1.253	0.043
11. I slept much more than usual.	1.463	0.090	0.052	1.027	0.086	1.715	0.135	2.220	0.178
12. I felt like I was moving too slowly.	2.947	0.028	0.030	0.625	0.034	1.107	0.049	1.554	0.071
13. I felt fidgety.	2.546	-0.036	0.034	0.493	0.035	0.988	0.049	1.342	0.066
14. I wished I were dead.	3.370	0.816	0.036	1.136	0.049	1.389	0.062	1.632	0.079
15. I wanted to hurt myself.	3.154	1.045	0.049	1.356	0.067	1.633	0.088	1.999	0.125
16. I was tired all the time.	2.999	-0.401	0.035	0.243	0.029	0.644	0.033	1.001	0.043
17. I did not like myself.	4.109	0.142	0.024	0.522	0.026	0.837	0.031	1.139	0.041
18. I lost a lot of weight without trying to.	1.087	1.521	0.175	2.190	0.257	2.753	0.331	3.278	0.403
19. I had a lot of trouble getting to sleep.	2.137	-0.255	0.043	0.493	0.041	0.987	0.056	1.307	0.071
20. I could not focus on the important things.	3.957	-0.073	0.026	0.434	0.026	0.859	0.032	1.199	0.043

*Note* GRM parameterization reported in Table 3 is discrimination*(theta – threshold)

### Comparing CESD-R Scores Using Percentiles

**Fig. 4:**
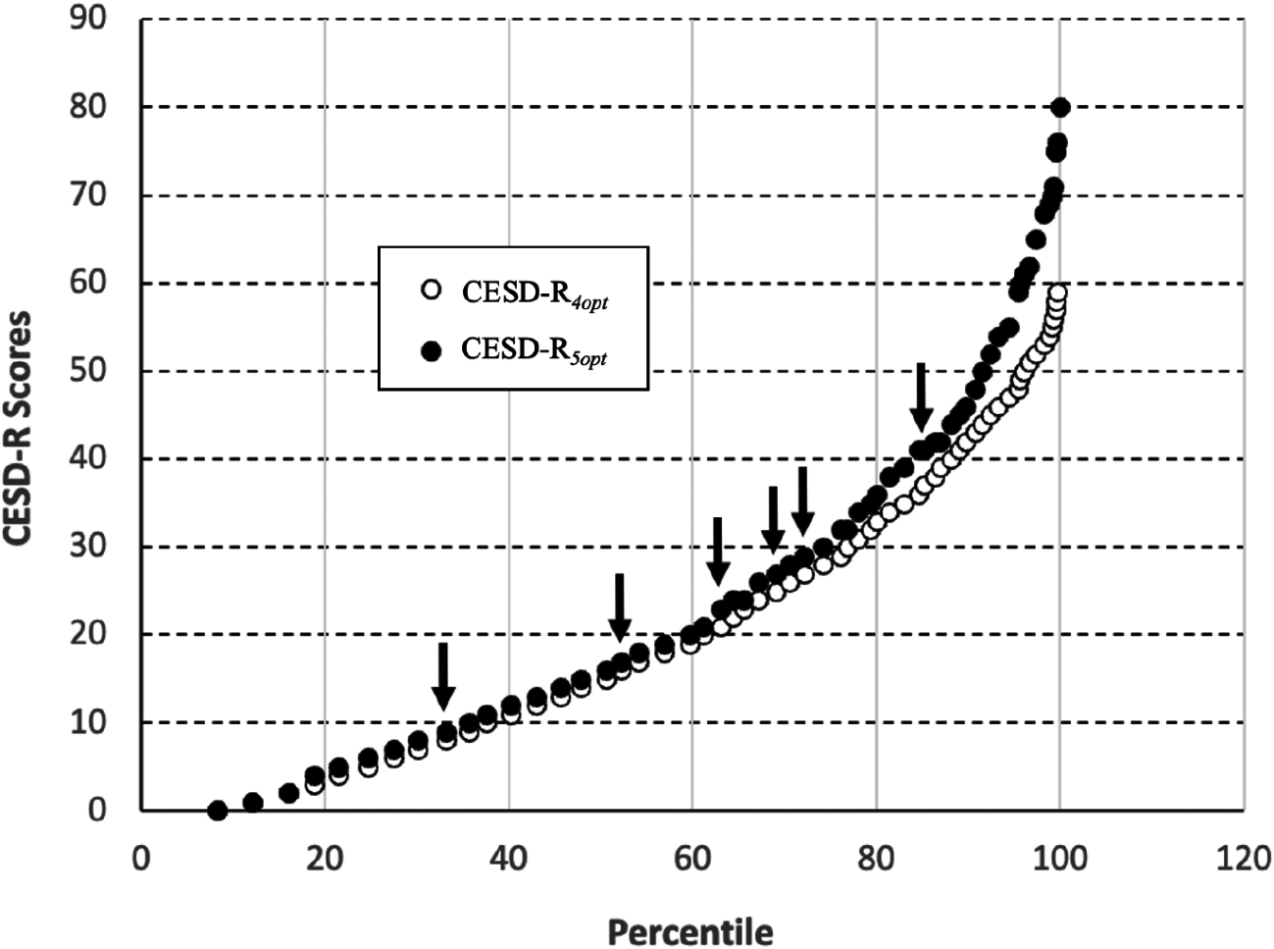
Percentiles corresponding to CESD-R_4opt_ and CESD-R_5opt_ Total Scores. *Note* The arrows indicate percentiles corresponding to scores of 8, 16, 21, 25, 27, and 37 on the CESD-R_4opt_ and scores of 9, 17, 23, 27, 29, and 41 on the CESD-R_5opt_

We calculated the percent of participants scoring at or below each sum score on both the CESD-R_4opt_ and CESD-R_5opt_. Matching these percentiles across the two measures enabled us to map CESD-R_4opt_ scores onto their corresponding scores on the CESD-R_5opt_ (as shown in Fig. 4). For participants with lower levels of depression, CESD-R_4opt_ scores and their corresponding CESD-R_5opt_ scores were nearly identical. At higher levels of depression, CESD-R_5opt_ scores accelerated faster than their corresponding CESD-R_4opt_ scores. For example, CESD-R_4opt_ scores of 8, 16, 21, 25, 27, and 37 corresponded to CESD-R_5opt_ scores of 9, 17, 23, 27, 29, and 41, respectively (see arrows in Fig. 4). Notably, scores of 16 and 27 are two of the more commonly used clinical cutoffs for depression for the CESD-R_4opt_ (Radloff, 1977; Gotlib et al., 1995; Kagee et al., 2020; Schulberg et al., 1985; Zich et al., 1990). These results suggest that users of the CESD-R_5opt_ should use clinical cutoff scores of 17 and 29 if they wish to capture the same level of clinical severity as scores of 16 and 27, respectively, on the CESD-R_4opt_.

## Discussion

In the current study, we conducted IRT analyses to determine whether scoring the CESD-R across the full range of possible scores from 0 to 80 using 5 response options per item (the CESD-R_5opt_) provides more information about a person’s underlying depression than collapsing item response options 3 and 4 in order to score the CESD-R on a scale from 0 to 60 using 4 response options per item (the CESD-R_4opt_), as is convention. The CESD-R_5opt_ provided nearly twice as much information about a person’s latent depression for individuals with higher levels of depression, whereas the CESD-R_4opt_ and CESD-R_5opt_ provided approximately equal amounts of information for participants at low to moderate levels of depression. Examinations of threshold estimates and item characteristic curves showed that responses of 4 (nearly every day over the past two weeks) indicated meaningfully greater experiences of any given depressive symptom than did responses of 3 (5–7 days over the past two weeks). Taken together, the results suggest that, as hypothesized, treating all response options separately and scoring the CESD-R on a 0 to 80 scale provides substantially more information about people’s depression than treating responses of 3 and 4 as equivalent in order to score the CESD-R on a 0 to 60 scale. Pending further validation, we recommend using a clinical cutoff score of 29 when scoring the CESD-R with the full range of scores. Although scoring the CESD-R on a 0 to 60 scale has been standard, such a practice needlessly throws away potentially useful information about people’s underlying levels of depression, especially for individuals with more severe depression.

Both the CESD-R_4opt_ and CESD-R_5opt_ demonstrated a unidimensional factor structure with similar factor loadings. Both CESD-R scoring versions also showed high and nearly identical estimates of test-level internal consistency. These results suggest that the CESD-R_5opt_ retains the established, strong psychometric properties of the CESD-R_4opt_. The overall pattern of results supports the assertion that CESD-R users can gain additional information on respondents’ depressive symptoms by choosing not to collapse response categories and instead scoring the CESD-R on a 0 to 80 scale without negatively impacting the strong psychometric properties of the CESD-R. Notably, scoring the CESD-R on a 0 to 60 scale requires the additional step of collapsing responses of 3 and 4 which can be skipped by retaining the full spectrum of response options when scoring. As such, the CESD-R_5opt_ is not only more informative than the CESD-R_4opt_ but is also easier to score. In this sample, percentile scores were approximately equal for participants reporting low to moderate levels of depression on both the CESD-R_5opt_ and CESD-R_4opt_, suggesting that, at low to moderate levels of depression, total scores on the CESD-R_4opt_ and CESD-R_5opt_ indicate similar levels of relative severity such that previously proposed clinical cutoff scores can continue to be used even with the CESD-R_5opt_. However, separation in percentile scores emerged at high levels of depression. Thus, researchers and clinicians interested in measuring depression across the full range of possible values, and not just at low or moderate levels, may benefit from the increased precision and nuance afforded by the CESD-R_5opt_ for individuals at higher levels of depression. The CESD-R_5opt_ may be especially well suited for distinguishing among highly depressed individuals and for tracking change in symptoms over time when the change from one assessment to the next may be small. It is critical for future research to replicate our results in treatment-seeking samples, as it is likely our ability to observe differences between scoring options was diminished by relatively low levels of depression in our sample. It is possible that IRT analyses conducted in treatment-seeking samples with greater depression may support the use of different clinical cutoffs between the CESD-R_4opt_ and CESD-R_5opt_.

The merits of the CESD-R_5opt_ will likely be greater in some situations than others. In applications where researchers and clinicians wish to quantify the severity of depressive symptoms in more seriously depressed individuals, the CESD-R_5opt_ has particular promise. For example, depression treatment outcome studies typically select participants who are clinically depressed and seek to quantify the reduction of depression levels in response to treatment (e.g., Francis et al., 2019). Using measures that are psychometrically stronger at such high levels of depression will increase the fidelity of these estimates and avoid unnecessary attenuation of treatment effect sizes (i.e., ceiling effects; Šimkovic & Träuble, 2019). Conversely, in applications where researchers or clinicians intend to screen people for depression (using CESD-R cutoffs) or seek to quantify depression severity in individuals with low levels of depression, the CESD-R_4opt_ will suffice (e.g., Pryor et al., 2016). Even in these applications, however, using the CESD-R_5opt_ has no downside.

The CESD-R_5opt_ is also well-positioned to support two notable trends in psychopathology research. First, the recognition that significant symptom heterogeneity exists across depressed individuals has led to calls to increase clinical and research focus on specific depressive symptoms and their interrelations (Fried & Nesse, 2015). Beyond improving the ability to track changes in depressive symptoms overall, the increased precision offered by the CESD-R_5opt_ over the CESD-R_4opt_ will likely extend to individual symptoms as well. Indeed, this supposition is supported by the item-level analyses, which showed that every item, except item 18, provided more information when scored on a 0 to 4 scale in the CESD-R_5opt_ compared to when scored on a 0 to 3 scale in the CESD-R_4opt_. Thus, researchers and clinicians interested in capturing information on specific depressive symptoms may find the CESD-R_5opt_ to be a useful improvement over the previous scoring approach to the CESD-R. Further, individual depressive symptoms such as difficulty concentrating and insomnia serve as transdiagnostic indicators of psychological dysfunction. The CESD-R_5opt_ may therefore facilitate assessment of transdiagnostic symptoms for research and clinical practice, at least relative to the CESD-R_4opt_.

Second, there has been increasing interest in moving away from categorical diagnostic approaches to mental disorders in favor of alternate nosological approaches that emphasize the use of transdiagnostic dimensions such as HiTOP (Kotov et al., 2021) and RDoC (Cuthbert, 2014). By far the two most popular diagnostic systems, the DSM-5 and the 11th revision of the International Classification of Diseases and Related Health Problems (ICD-11; World Health Organization, 2019) have faced calls to broadly incorporate dimensionality into their classification schemes (Gaebel et al., 2020; Shankman et al., 2018). Some limited dimensionality in the diagnosis of depressive disorders currently exists within both the DSM-5 and the ICD-11, specifically the current severity specifiers of mild, moderate, and severe. The CESD-R has always offered the ability to quantify the severity of depression dimensionally; however, the increased precision offered by the CESD-R_5opt_ at high levels of depression enables the scale to more accurately measure the entire spectrum of depression severity. Administration of the CESD-R_5opt_ may also assist researchers and clinicians in distinguishing between current severity specifiers, especially between moderate and severe depression.

Various limitations of the current study suggest additional avenues for future research. The generalizability of study results is limited by the predominantly White and female nature of the study sample. As such, future research should examine ways the CESD-R_4opt_ and CESD-R_5opt_ may operate differently in more diverse samples. Additionally, recent research suggests that there may be qualitative distinctions between subtypes of depression based on the length and chronicity of depressive episodes. Specifically, Klein and colleagues (2023) conducted a detailed, interview-based assessment of depressive history and course, identifying two depressive subtypes. These subtypes, chronic-intermittent depression and time-limited depression, differed based on the total amount of time spent in depression across the lifetime and showed differential associations with several well-supported predictors of depression onset and severity. We are unaware of any studies that have administered the CESD-R to both subtypes, so it is unknown whether the CESD-R has equivalent psychometric properties across these groups. This uncertainty extends to whether the CESD-R_5opt_ would provide more information than the CESD-R_4opt_ for each of these subgroups. Finally, the current study did not formally compare potential CESD-R_5opt_ clinical cutoff scores to actual diagnoses. Although our data suggest that cutoffs for the CESD-R_4opt_ and CESD-R_5opt_ are similar, further validation is needed.

Altogether, the IRT analyses conducted in the current study provide preliminary evidence to suggest that, contrary to convention, the CESD-R should be scored along the entire possible range of scores, from 0 to 80 using all 5 response options per item. This treats reports of experiencing symptoms for 5–7 days over the past two weeks as meaningfully different indicators of depressive severity than reports of experiencing symptoms nearly every day over the past two weeks. In so doing, researchers and clinicians gain additional information about people’s underlying levels of depression, especially for individuals who are more severely depressed, without any penalty to psychometric performance or scoring efficiency. Additional research is needed to replicate these results, particularly studies using treatment-seeking samples and diagnostic interviews to establish clinical cutoff scores at various levels of depressive severity for the CESD-R_5opt_.

## Electronic Supplementary Material

Below is the link to the electronic supplementary material.

Supplementary Material 1

## Data Availability

The data and analysis code supporting the results reported in this study are available upon reasonable request.
