# Supplementary material for: Improved Scoring of the Center for Epidemiologic Studies Depression Scale – Revised: An Item Response Theory Analysis
Source: J Psychopathol Behav Assess. 2024 Jul 16;46(3):783–92. doi: 10.1007/s10862-024-10155-y (PMC11447136; doi:10.1007/s10862-024-10155-y)
Supplement: Supplementary file 1 — Supplementary Material 1 [file 10862_2024_10155_MOESM1_ESM.docx]

Improved Scoring of the Center for Epidemiologic Studies Depression Scale – Revised:

An Item Response Theory Analysis

Supplementary Material

**Table S1**

$\chi^{2}$*Likelihood Ratio Tests for DIF Analysis*

| Item | CESD-R_4opt_ | | | CESD-R_5opt_ | | |
| --- | --- | --- | --- | --- | --- | --- |
|  | Uniform DIF | Non-uniform DIF | Total DIF | Uniform DIF | Non-uniform DIF | Total DIF |
| 1. My appetite was poor. | 0.000 | 0.000 | 0.000 | 0.000 | 0.000 | 0.000 |
| 2. I could not shake off the blues. | 0.159 | 0.029 | 0.034 | 0.179 | 0.017 | 0.024 |
| 3. I had trouble keeping my mind on what I was doing. | 0.000 | 0.000 | 0.000 | 0.000 | 0.000 | 0.000 |
| 4. I felt depressed. | 0.000 | 0.049 | 0.000 | 0.000 | 0.272 | 0.000 |
| 5. My sleep was restless. | 0.000 | 0.002 | 0.000 | 0.000 | 0.011 | 0.000 |
| 6. I felt sad. | 0.130 | 0.703 | 0.296 | 0.182 | 0.976 | 0.409 |
| 7. I could not get going. | 0.003 | 0.780 | 0.012 | 0.006 | 0.621 | 0.020 |
| 8. Nothing made me happy. | 0.000 | 0.255 | 0.000 | 0.000 | 0.416 | 0.000 |
| 9. I felt like a bad person. | 0.685 | 0.042 | 0.115 | 0.615 | 0.066 | 0.163 |
| 10. I lost interest in my usual activities. | 0.000 | 0.381 | 0.000 | 0.000 | 0.164 | 0.000 |
| 11. I slept much more than usual. | 0.683 | 0.827 | 0.898 | 0.713 | 0.727 | 0.880 |
| 12. I felt like I was moving too slowly. | 0.944 | 0.102 | 0.261 | 0.959 | 0.095 | 0.247 |
| 13. I felt fidgety. | 0.000 | 0.004 | 0.000 | 0.001 | 0.004 | 0.000 |
| 14. I wished I were dead. | 0.060 | 0.581 | 0.147 | 0.059 | 0.805 | 0.164 |
| 15. I wanted to hurt myself. | 0.265 | 0.718 | 0.503 | 0.417 | 0.576 | 0.615 |
| 16. I was tired all the time. | 0.250 | 0.331 | 0.322 | 0.333 | 0.725 | 0.589 |
| 17. I did not like myself. | 0.192 | 0.343 | 0.272 | 0.323 | 0.130 | 0.195 |
| 18. I lost a lot of weight without trying to. | 0.000 | 0.808 | 0.001 | 0.000 | 0.703 | 0.001 |
| 19. I had a lot of trouble getting to sleep. | 0.000 | 0.001 | 0.000 | 0.000 | 0.003 | 0.000 |
| 20. I could not focus on the important things. | 0.001 | 0.004 | 0.000 | 0.000 | 0.048 | 0.000 |

*Note***.** The numbers reported are *p*-values.

**Table S2**

*McFadden’s Pseudo* $R^{2}$ *Effect Sizes for DIF Analysis*

| Item | CESD-R_4opt_ | | | CESD-R_5opt_ | | |
| --- | --- | --- | --- | --- | --- | --- |
|  | Uniform DIF | Non-uniform DIF | Total DIF | Uniform DIF | Non-uniform DIF | Total DIF |
| 1. My appetite was poor. | 0.020 | 0.008 | 0.028 | 0.019 | 0.007 | 0.027 |
| 2. I could not shake off the blues. | 0.001 | 0.002 | 0.003 | 0.001 | 0.002 | 0.003 |
| 3. I had trouble keeping my mind on what I was doing. | 0.023 | 0.006 | 0.029 | 0.019 | 0.006 | 0.026 |
| 4. I felt depressed. | 0.009 | 0.002 | 0.011 | 0.010 | 0.001 | 0.010 |
| 5. My sleep was restless. | 0.009 | 0.004 | 0.013 | 0.008 | 0.003 | 0.010 |
| 6. I felt sad. | 0.001 | 0.000 | 0.001 | 0.001 | 0.000 | 0.001 |
| 7. I could not get going. | 0.004 | 0.000 | 0.004 | 0.003 | 0.000 | 0.003 |
| 8. Nothing made me happy. | 0.022 | 0.001 | 0.023 | 0.021 | 0.000 | 0.021 |
| 9. I felt like a bad person. | 0.000 | 0.002 | 0.002 | 0.000 | 0.002 | 0.002 |
| 10. I lost interest in my usual activities. | 0.009 | 0.000 | 0.010 | 0.010 | 0.001 | 0.011 |
| 11. I slept much more than usual. | 0.000 | 0.000 | 0.000 | 0.000 | 0.000 | 0.000 |
| 12. I felt like I was moving too slowly. | 0.000 | 0.001 | 0.001 | 0.000 | 0.001 | 0.001 |
| 13. I felt fidgety. | 0.006 | 0.004 | 0.009 | 0.005 | 0.003 | 0.008 |
| 14. I wished I were dead. | 0.003 | 0.000 | 0.003 | 0.003 | 0.000 | 0.003 |
| 15. I wanted to hurt myself. | 0.001 | 0.000 | 0.001 | 0.001 | 0.000 | 0.001 |
| 16. I was tired all the time. | 0.001 | 0.000 | 0.001 | 0.000 | 0.000 | 0.000 |
| 17. I did not like myself. | 0.001 | 0.000 | 0.001 | 0.000 | 0.001 | 0.001 |
| 18. I lost a lot of weight without trying to. | 0.012 | 0.000 | 0.012 | 0.011 | 0.000 | 0.011 |
| 19. I had a lot of trouble getting to sleep. | 0.016 | 0.005 | 0.020 | 0.014 | 0.004 | 0.018 |
| 20. I could not focus on the important things. | 0.005 | 0.004 | 0.009 | 0.006 | 0.002 | 0.008 |

**Table S3**

*RMSEA of GRM Results for CESD-R_4opt_ and CESD-R_5opt_*

| Item | CESD-R_4opt_ | CESD-R_5opt_ |
| --- | --- | --- |
| 1. My appetite was poor. | 0.000 | 0.000 |
| 2. I could not shake off the blues. | 0.010 | 0.007 |
| 3. I had trouble keeping my mind on what I was doing. | 0.010 | 0.000 |
| 4. I felt depressed. | 0.015 | 0.008 |
| 5. My sleep was restless. | 0.012 | 0.013 |
| 6. I felt sad. | 0.003 | 0.000 |
| 7. I could not get going. | 0.012 | 0.012 |
| 8. Nothing made me happy. | 0.016 | 0.023 |
| 9. I felt like a bad person. | 0.000 | 0.013 |
| 10. I lost interest in my usual activities. | 0.012 | 0.010 |
| 11. I slept much more than usual. | 0.004 | 0.019 |
| 12. I felt like I was moving too slowly. | 0.000 | 0.008 |
| 13. I felt fidgety. | 0.012 | 0.000 |
| 14. I wished I were dead. | 0.014 | 0.000 |
| 15. I wanted to hurt myself. | 0.022 | 0.007 |
| 16. I was tired all the time. | 0.000 | 0.000 |
| 17. I did not like myself. | 0.000 | 0.000 |
| 18. I lost a lot of weight without trying to. | 0.016 | 0.020 |
| 19. I had a lot of trouble getting to sleep. | 0.000 | 0.011 |
| 20. I could not focus on the important things. | 0.010 | 0.000 |

*Note*. RMSEA = Root mean square error of approximation.

**Figure S1a**

*Comparison of Samples for All Items Using CESD-R_4opt_*


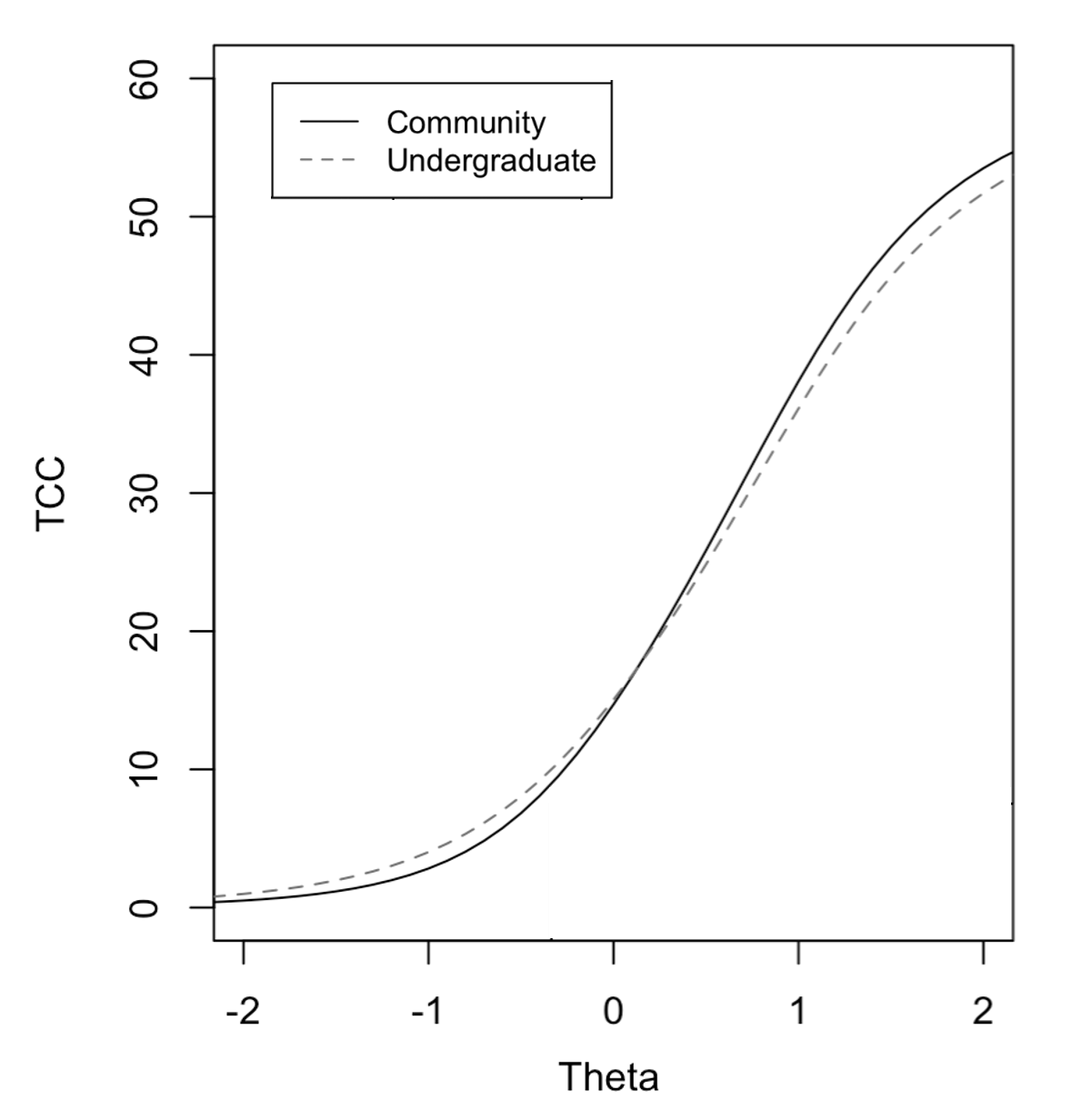


*Note*. TCC = Test characteristic curve.

**Figure S1b**

*Comparison of Samples for DIF Items Using CESD-R_4opt_*


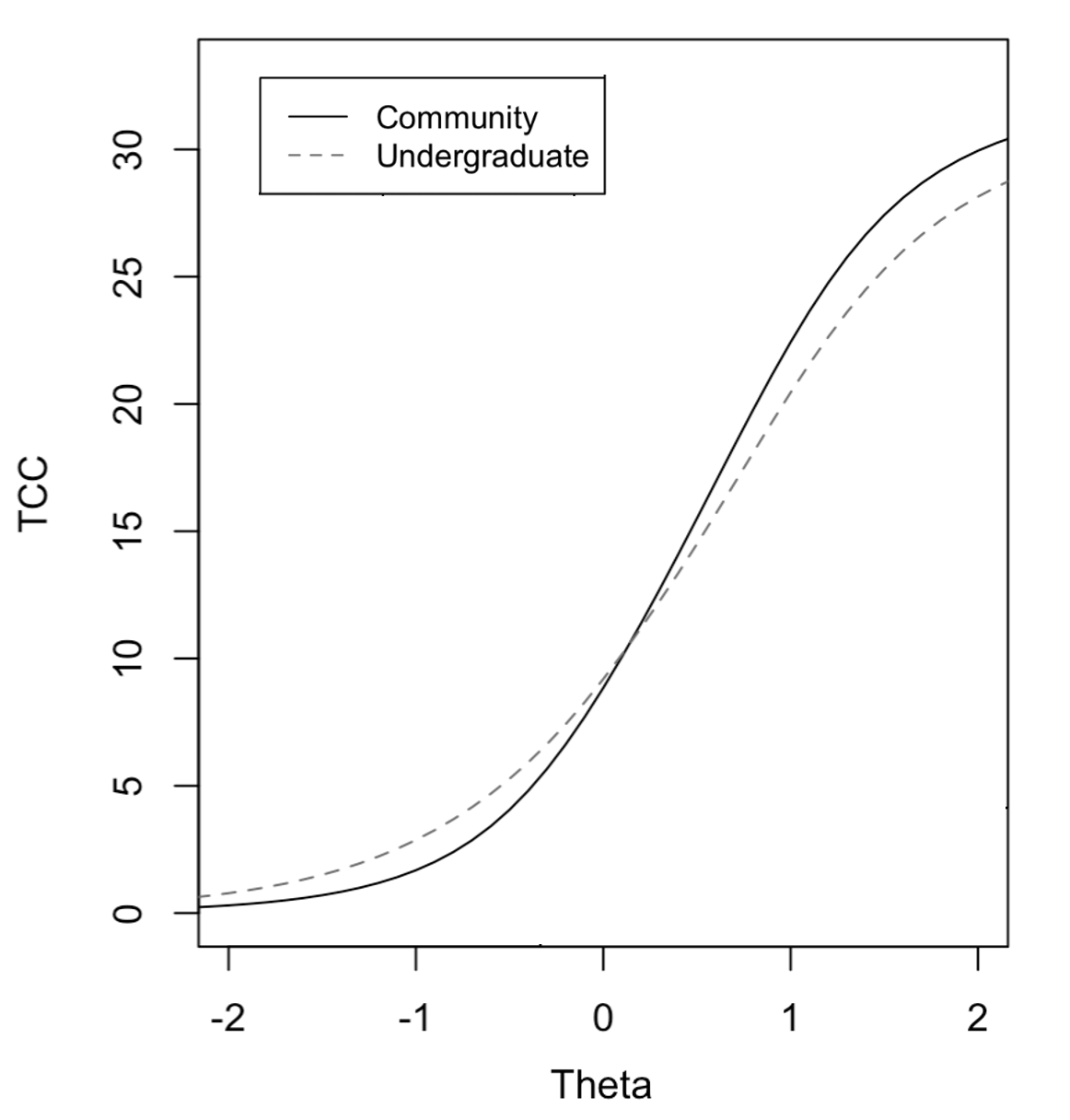


*Note*. TCC = Test characteristic curve.

**Figure S1c**

*Comparison of Samples for All Items Using CESD-R_5opt_*


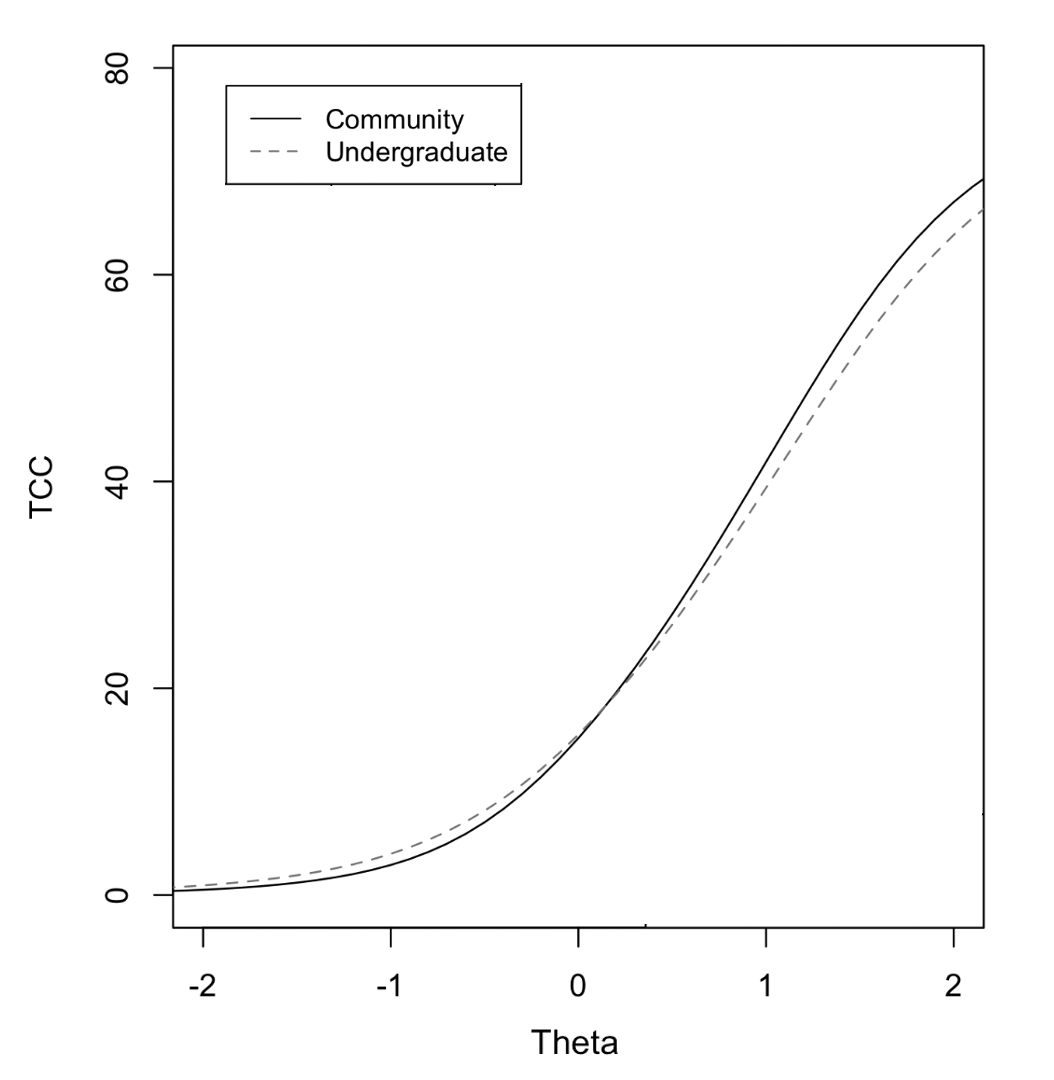


*Note.* TCC = Test characteristic curve.

**Figure S1d**

*Comparison of Samples for DIF Items Using CESD-R_5opt_*


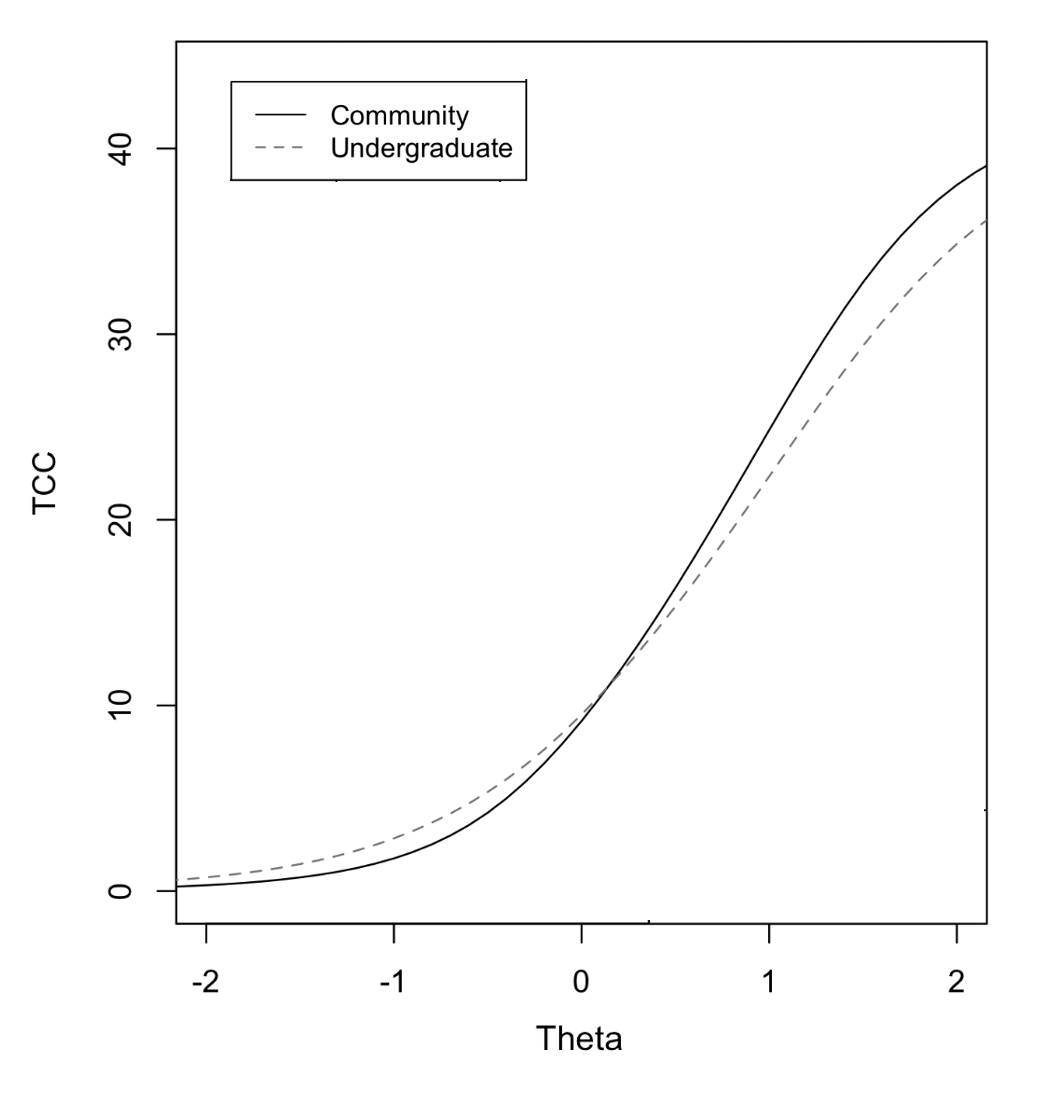


*Note.* TCC = Test characteristic curve.

**Figure S2a**

*Parallel Analysis for CESD-R_4opt_*


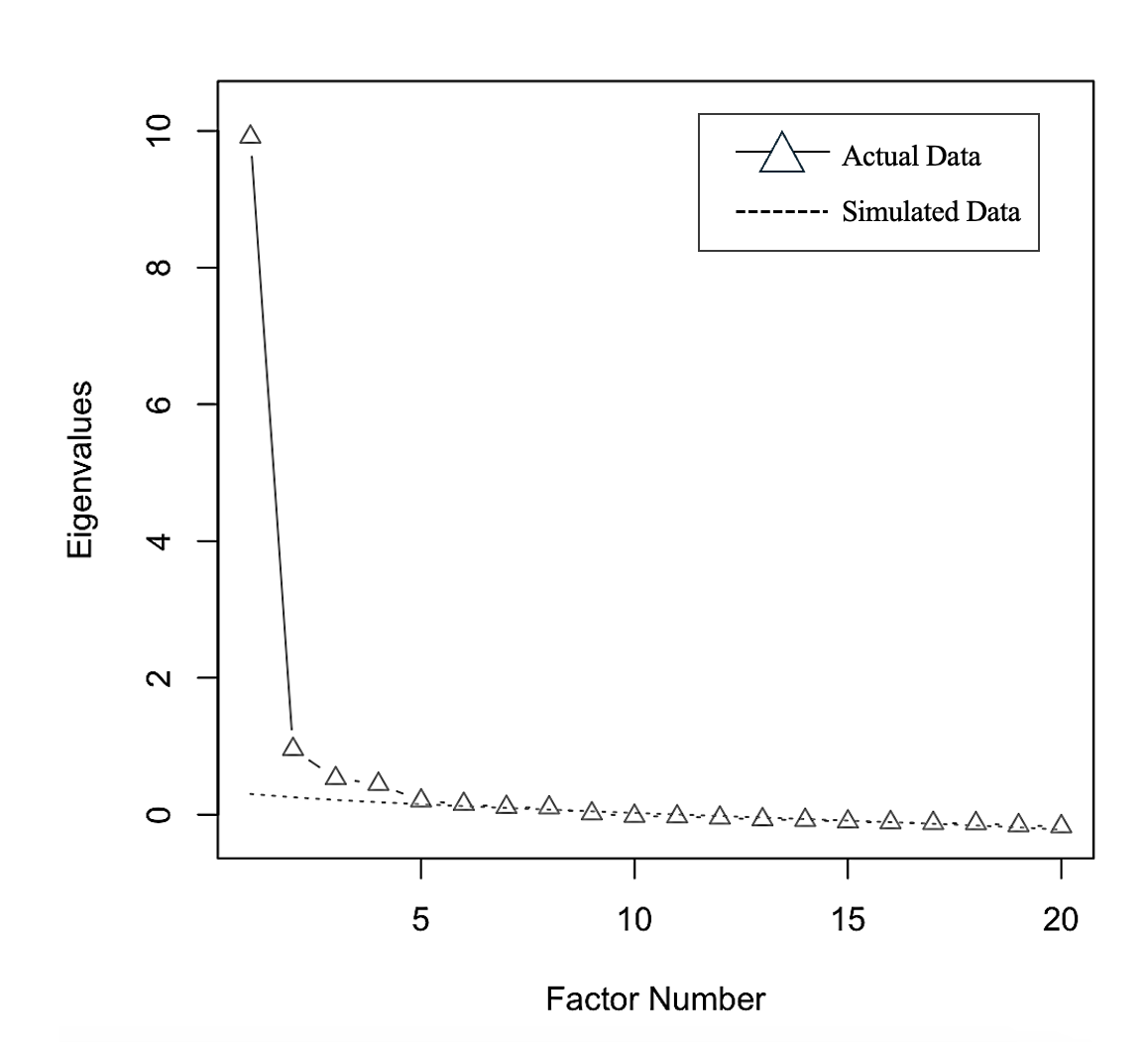


**Figure S2b**

*Parallel Analysis for CESD-R_5opt_*

**
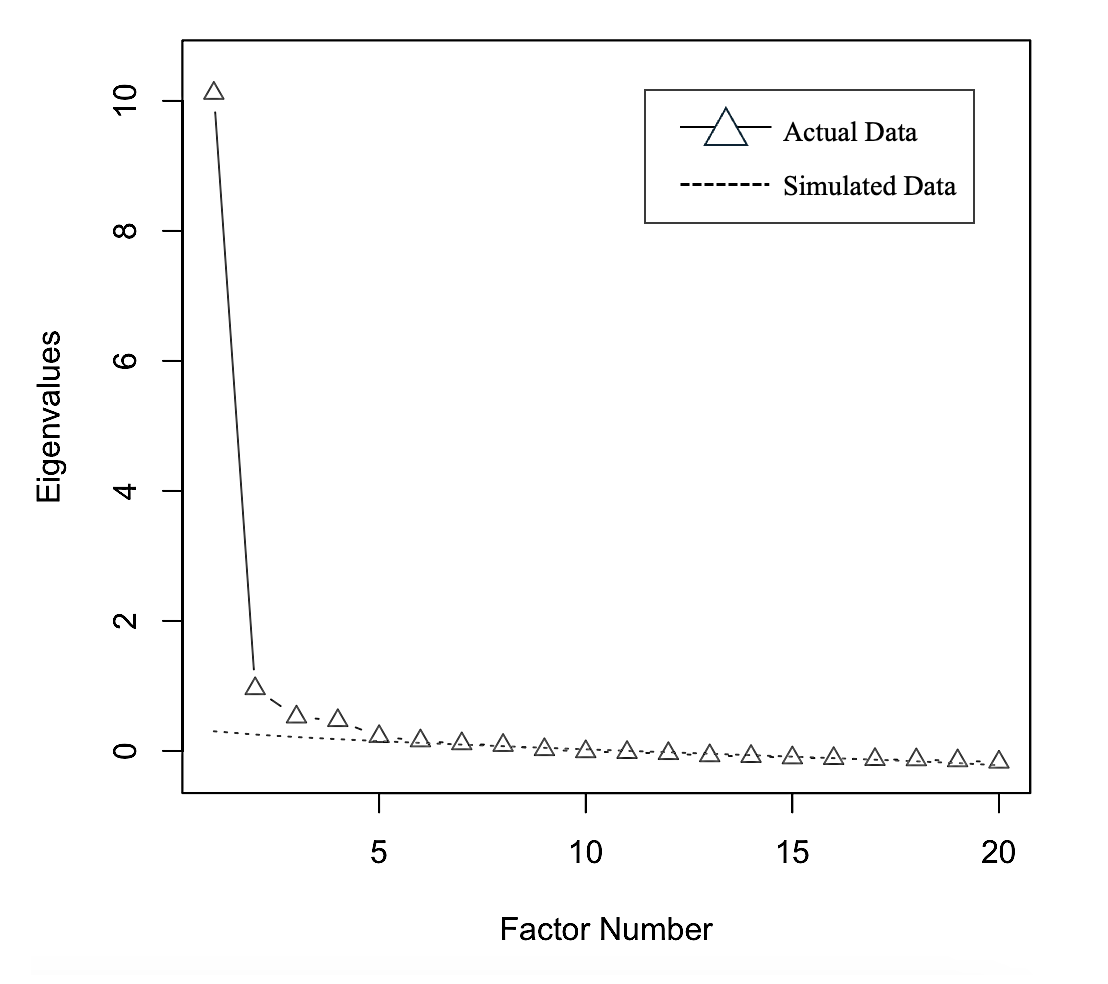
**

**Figure S3**

*Relative Efficiency Curves Describing the Ratio of Item Information for CESD-R_5opt_ to that of CESD-R_4opt_*

*
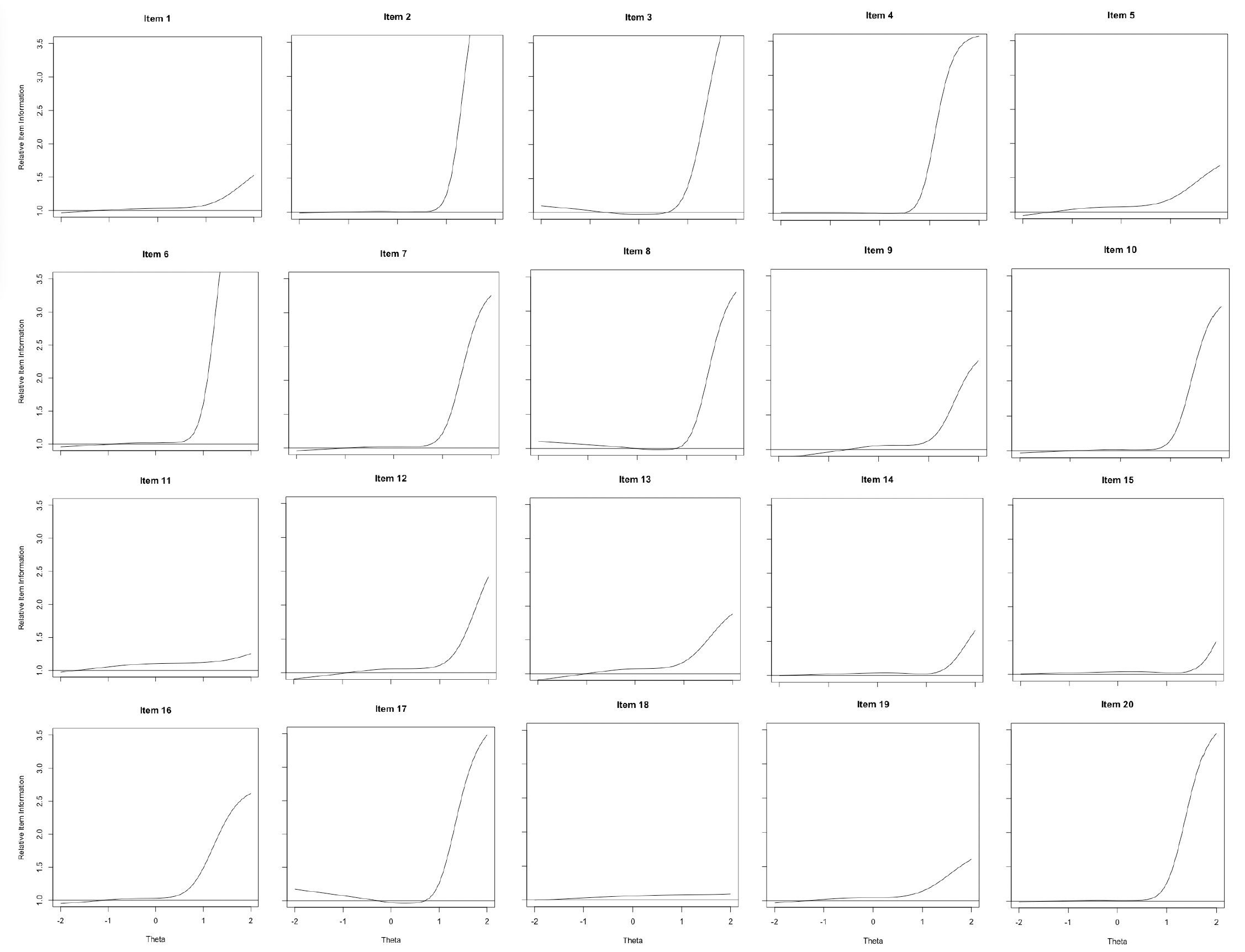
*

**Figure S4**

*Item Characteristic Curves of Graded Response Model*


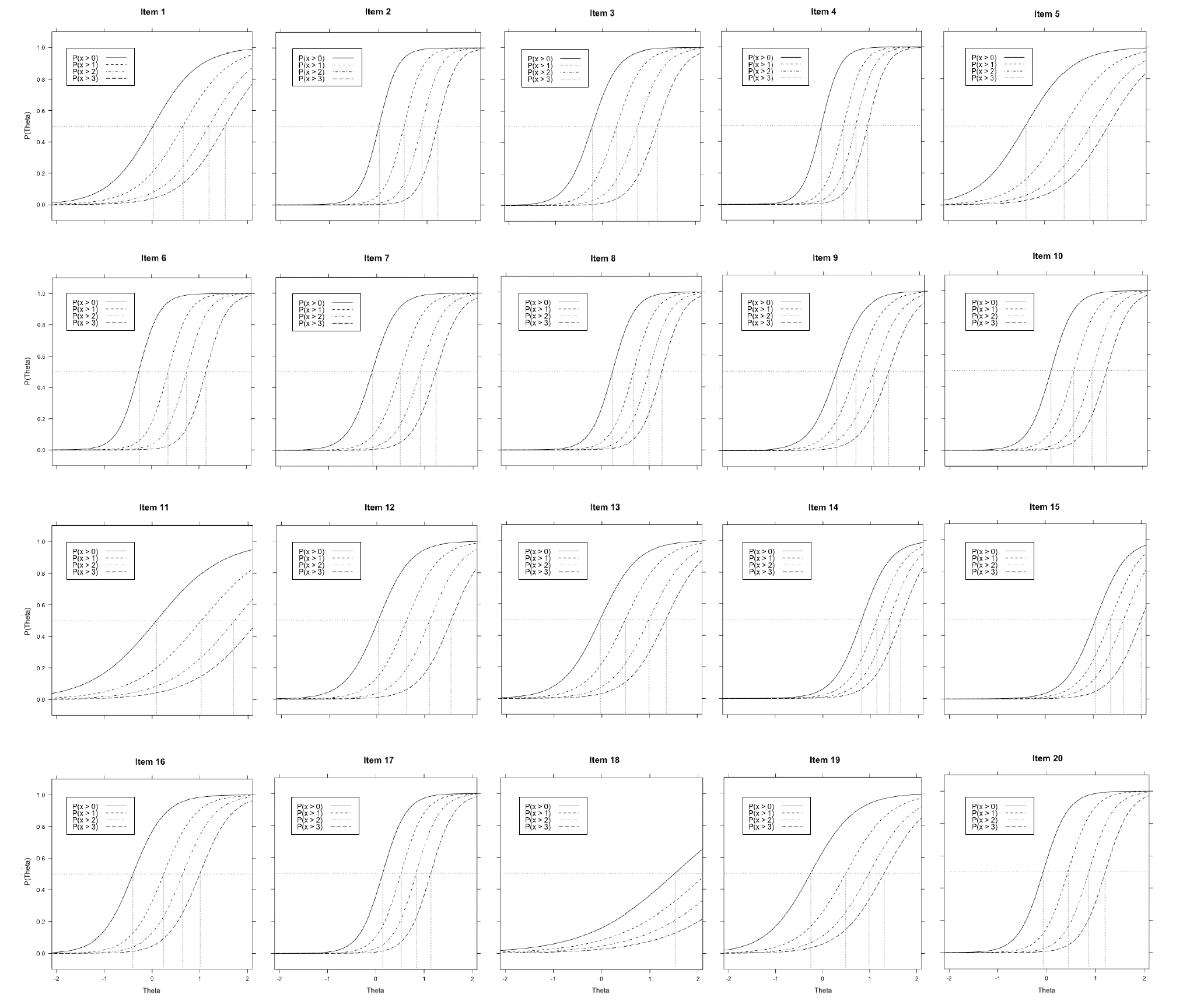


*Note*. The x- and y-axes are the same for each item. P(x > 0) indicates the probability of having a score above 0; P(x > 1) indicates the probability of having a score above 1; P(x > 2) indicates the probability of having a score above 2; P(x > 3) indicates the probability of having a score above 3. Threshold estimates for each item correspond to the “theta” points at which each probability equals 0.5, as noted in Table 3.
